# Pulmonary Hypertension Associated with Idiopathic Pulmonary Fibrosis: Current and Future Perspectives

**DOI:** 10.1155/2017/1430350

**Published:** 2017-02-13

**Authors:** Scott D. Collum, Javier Amione-Guerra, Ana S. Cruz-Solbes, Amara DiFrancesco, Adriana M. Hernandez, Ankit Hanmandlu, Keith Youker, Ashrith Guha, Harry Karmouty-Quintana

**Affiliations:** ^1^Department of Biochemistry and Molecular Biology, University of Texas Medical School at Houston, Houston, TX 77030, USA; ^2^Methodist DeBakey Heart and Vascular Center, The Methodist Hospital, Houston, TX 77030, USA; ^3^Methodist J.C. Walter Jr. Transplant Center, The Methodist Hospital, Houston, TX 77030, USA

## Abstract

Pulmonary hypertension (PH) is commonly present in patients with chronic lung diseases such as Chronic Obstructive Pulmonary Disease (COPD) or Idiopathic Pulmonary Fibrosis (IPF) where it is classified as Group III PH by the World Health Organization (WHO). PH has been identified to be present in as much as 40% of patients with COPD or IPF and it is considered as one of the principal predictors of mortality in patients with COPD or IPF. However, despite the prevalence and fatal consequences of PH in the setting of chronic lung diseases, there are limited therapies available for patients with Group III PH, with lung transplantation remaining as the most viable option. This highlights our need to enhance our understanding of the molecular mechanisms that lead to the development of Group III PH. In this review we have chosen to focus on the current understating of PH in IPF, we will revisit the main mediators that have been shown to play a role in the development of the disease. We will also discuss the experimental models available to study PH associated with lung fibrosis and address the role of the right ventricle in IPF. Finally we will summarize the current available treatment options for Group III PH outside of lung transplantation.

## 1. Prevalence of PH in IPF

Chronic lung diseases including Chronic Obstructive Pulmonary Disease (COPD) and Idiopathic Pulmonary Fibrosis (IPF) represent the third leading cause of death in the US [1]. Despite reductions in mortality in cancer and cardiovascular disease, mortality rates for chronic lung diseases have remained unaffected over the last decade [2]. An important complication of chronic lung diseases that is strongly linked to the mortality is the presence of pulmonary hypertension (PH) [3, 4].

PH is defined by a mean pulmonary arterial pressure (mPAP) of ≥25 mmHg and a pulmonary artery wedge pressure (PAWP) of ≤15 mmHg and elevated pulmonary vascular resistance (PVR) > 3 wood units (WU) [5]. The pathologic process in PH is characterized by extensive vascular remodeling, including enhanced proliferation of pulmonary artery smooth muscle cells (PASMC). This leads to narrowing and obliteration of the vessel lumen resulting in increased vascular tone. Similarly, due to the increased pressure load in the pulmonary vasculature, the right ventricle (RV) tries to compensate resulting in remodeling, hypertrophy, dysfunction, and eventually right-sided heart failure and death [6–8]. PH is subdivided into 5 comprehensive subsets of PH: Group I–Group V PH. Group I PH includes Pulmonary Arterial Hypertension (PAH) of idiopathic or heritable origin where the lung vasculature is affected but not the lung parenchyma. Group II PH is associated with left heart disease. Group III PH is associated with chronic lung diseases affecting the lung parenchyma and hypoxemia. Group IV PH is chronic thromboembolic pulmonary hypertension (CTEPH); finally Group V PH includes PH from unclear and multifactorial mechanisms [9]. This review will focus on Group III PH. Group III PH is associated with chronic lung diseases such as Chronic Obstructive Pulmonary Disease (COPD) and IPF [3, 4]. In this review article we will focus on PH associated with IPF. IPF is a parenchymal disease of the lung, manifested by chronic cough, exertional dyspnea, and often leading to acute respiratory failure [10]. The prevalence of IPF is estimated to be in the range of 14–43 per 100,000 persons, with the range dependent upon the criteria applied [11, 12]. It important to mention that IPF is a fatal disease that affects millions of people worldwide, yet the funding for chronic respiratory diseases desperately lags behind its societal burden [2].

The prevalence of PH amongst IPF patients is dependent upon the severity of IPF. In the early stages or when initially diagnosed, PH affects < 10% of IPF patients [4, 13]. However, as IPF advances, the incidence of PH increases markedly. One study of patients awaiting lung transplant, and thus in an advanced stage of IPF, reported an incidence of 32% [14]. Subsequent studies have largely supported or increased this percentage to be between 32 and 50% [12, 15]. However, it is important to mention that the symptoms for PH and IPF are very similar (shortness of breath and exertional dyspnea) and as such it is conceivable for PH to be underdiagnosed in patients with IPF. Furthermore, typically the mPAP of patients with IPF is significantly lower than patients with Group I PH [16–19]. Despite this, the presence of PH in IPF is strongly linked to the increased mortality [12, 14] and efforts aimed at treating PH in IPF may provide much needed therapies to diminish the mortality of IPF.

## 2. Epigenetics of PH Associated with IPF

The role of epigenetics in the development of IPF is an intense area of research [20–23]. Similarly, there is a vast literature that has looked at epigenetic changes in Group I PH, namely, PAH [24–26]. These studies have focused on IPF or PAH with little or no research into Group III PH and in particular to PH associated with IPF. Thus, there is a strong need for enhanced research in this niche, as increased knowledge regarding epigenetic changes in patients with IPF and PH is necessary not only to develop new therapies but also to enable personalized care for patients with IPF afflicted with PH. Important lessons that can be applied to IPF and PH are how microRNAs (miRNAs) through transcriptional and translational regulation have the capacity to influence target genes in the development of PAH [25]. From the field of IPF, DNA methylation by DNA methyltransferases has been shown to impact myofibroblast differentiation through regulation of *α*SMA expression in developmental IPF [27]. Importantly environmental factors such as cigarette smoke have been implicated in manipulating the methylome and the methylation patterns of specific promoters in genes that are involved in IPF [28, 29]. Although these mechanisms have not explicitly been identified in PH associated with IPF, it is conceivable that similar processes may be unique in patients in IPF and PH that can provide insight into the pathogenesis of PH in the setting of IPF in addition to providing unique therapeutic avenues for Group III PH.

## 3. Mediators of PH in IPF

**Figure 1:**
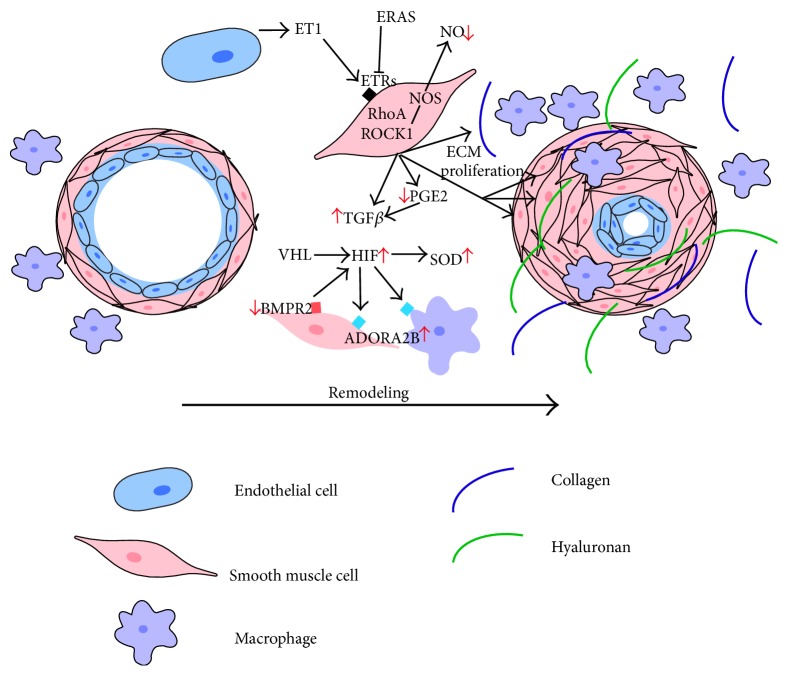
Cellular processes and mediators involved in the pathogenesis of PH associated with lung fibrosis.

The general consensus regarding the pathogenesis of PH in IPF was that the development of PH was a complication of the unrelenting fibroproliferative injury in IPF and that treating the underlying lung fibrosis would also treat PH. Although many animal models have indeed shown that treating lung fibrosis results in attenuation of hallmarks of PH, current therapies for IPF are unable to reverse the fibroproliferative damage in IPF. This observation and adverse independent impact of PH in these patients highlight the need to develop therapies that directly impact PH in IPF. In order to achieve this aim we must understand the pathogenesis of PH in IPF. Although chronic hypoxia has been deemed integral in the development of Group III PH as a whole, the downstream mechanisms that lead to PH in IPF are not fully understood. Many diverse mediators including endothelin 1 (ET-1), transforming growth factor- (TGF-) *β*, prostaglandin (PG) E2, bone morphogenetic protein receptor type 2 (BMPR2), adenosine signaling, hyaluronan, and IL-6 have been shown to play important roles in both the progression of fibrosis and the development of PH. However, how these mediators may contribute independently to the pathogenesis of PH in the context of lung fibrosis is not fully understood.  Figure 1 summarizes some of the key mediators in PH associated with IPF.

ET-1 is a strong vasoconstrictor that has been implicated in both Group I PH and pulmonary fibrosis. ET-1 was found to be produced in the vascular endothelium of patients with both PH and lung fibrosis but not in normal lung or in those patients solely with fibrosis [30]. This increase in ET-1 levels was also seen in the plasma of patients with Group I PH and was correlated with increased mPAP [31]. ET-1 expression is also elevated in the airway epithelium of patients with IPF [32]. ET-1 acts in several pathways; canonically it binds two ET receptors on vascular smooth muscle cells activating calcium signaling and vasoconstriction; ET-1 can also stimulate proliferation, as well as growth factor and extracellular matrix production [33–36]. The endothelin receptor antagonists bosentan and macitentan have been shown to reduce both the fibroproliferative injury and PH in the rat bleomycin model of pulmonary fibrosis with PH [37]. This also correlated with an increased exercise capacity [38].

A potential activator of ET-1, the RhoA pathway, and downstream effectors Rho-kinases I and II (ROCKs) have been associated with the development of PH in several animal models including bleomycin, hypoxia, and monocrotaline [39–43]. In fact, treatment of the mouse bleomycin model of pulmonary fibrosis and PH with fasudil, a selective ROCK inhibitor, not only reduced the PH hallmarks of vascular remodeling and right ventricle systolic pressure but also reduced lung fibrosis [40]. ROCKs are involved in many pathways involved in the development of PH and fibrosis including proliferation, survival, migration, and contraction [44]. Interestingly, inhibition of the RhoA/ROCK pathway is effective in those animal models that are resistant to inhaled nitric oxide (NO) treatment, a phenomenon that is common in patients with PH associated with IPF that do not respond to NO treatment [45]. This pathway has also been shown to play a role in Group I PH where ROCK inhibitors appear to have therapeutic value [46, 47]. PDE5A inhibitors also can attenuate fibrosis associated PH both in patients and in the murine bleomycin model; this attenuation proceeds through the reduction of ROS and the RhoA/ROCK pathway [48, 49].

TGF-*β* is one of the most potent profibrotic cytokines; during disease states such as IPF or PH, TGF-*β* signaling can induce proliferation of vascular smooth muscle cells and increased production of extracellular matrix (ECM) components [50, 51]. TGF-*β* signaling proceeds through two pathways, canonically through Smad7 to activate the Smad2/3 and Smad4 binding to activate transcription and a Smad independent pathway that proceeds through the p38 MAPK and JNK [52, 53]. Several processes can cause abnormal TGF-*β* signaling; 5-lipoxygenase can shunt precursors of PGE2, an antifibrogenic factor, causing reduced expression while cellular damage to epithelial cells can also reduce the production of PGE2 [54]. This reduction of PGE2 can lead to increased TGF-*β* production [55]. A second pathway that can result in TGF-*β* signaling is the loss of signaling through the bone morphogenetic protein receptor 2 (BMPR2). BMPR2 is a member of the TGF-*β* superfamily and has a major role in suppressing TGF-*β* signaling in normal tissues. BMPR2 signaling activates Smad1/5/8 activation which directly acts to inhibit Smad2/3 signaling, thus reducing the downstream effects of TGF-*β* activation [56]. BMPR2 levels are inversely correlated with mPAP in patients with IPF and reduced BMPR2 expression can cause fibroproliferation [57]. Expression of mutated BMPR2 in an experimental model of lung fibrosis and PH shows increased PH compared to wild type mice [58]. These studies show that reduced BMPR2 may contribute to both fibrosis and PH through excessive TGF-*β* signaling.

Another role that TGF-*β* may play in Group III PH is through promoting endothelial to mesenchymal transition (EndMT) [59, 60]. EndMT includes a change in cell shape, loss of contact inhibition, and an increase in motility related to increased ECM production. This transition, in addition to endothelial cell injury, can contribute to fibrosis and could also explain the reduced effectiveness of endothelin receptor antagonist, a group of oral vasodilators including macitentan and bosentan that show little effectiveness in Group III PH [61].

Interestingly, expression of mutant BMPR2 in mice resulted in increased hypoxia inducible factor (HIF)1A stabilization allowing for activation of HIF inducible transcripts [58]. The HIF pathway has been shown to play an important role in the progression of several chronic lung diseases including IPF. In the bleomycin model of lung fibrosis transgenic mice with HIF deleted in the vascular endothelial cells were protected from PH while still developing fibrosis. Aberrant HIF signaling can promote PASMC and permeability contributing to PH progression [62]. HIF signaling in normoxic conditions is suppressed by the tumor suppressor von Hippel-Lindau (VHL) [63]. Patients carrying a VHL mutation have many symptoms including cancerous and noncancerous tumors but also PH. In transgenic mice with a mutated VHL protein, hallmarks of PH and lung fibrosis have been documented. This suggest a mechanistic link between HIF signaling and PH in IPF [64].

While hypoxia plays a role in the development of IPF and PH, reactive oxygen species can also contribute to the development of these diseases. Superoxide dismutase (SOD) is an enzyme that catalyzes the transition of a superoxide anion to oxygen or hydrogen peroxide relieving the oxidative potential of this species. It has been shown in the TGF-*β* overexpression rat model of pulmonary fibrosis that overexpression of extracellular SOD (EC-SOD) is protective against the fibrosis caused by the excess TGF-*β* signaling which can be activated by ROS [65]. Although strictly not in a model of lung fibrosis and PH, the depletion of EC-SOD leads to increased vascular remodeling in bleomycin-induced bronchopulmonary dysplasia (BPD), where features of PH are evident [66]. Also, the specific KO of EC-SOD in PASMC shows increased PH in the hypoxia model mouse model of PH [67]. Taken together these findings support the role of EC-SOD in both IPF and PH.

Similar to the role of EC-SOD in the rat bleomycin model of BPD and PH inhibition of tissue necrosis factor (TNF-*α*) signaling by both pharmaceutical intervention and exposure to 7% CO_2_ caused reduced PH symptoms and reduced lung injury, indicating a role of TNF-*α* signaling in disease development [68]. Additionally, thiol-redox alterations have been shown to be induced during in vitro exposure of lung vascular endothelial cells to bleomycin. These changes are accompanied by morphological and physiological changes along with increased ROS associated with vascular remodeling during pulmonary fibrosis that are abrogated by treatment with thiol-redox protective reagents [69]. Linking TGF-*β* and ET-1 signaling and these oxidative stress pathways is the nitric oxide synthase pathway. Nitric oxide synthase (NOS) produces NO which is a vasodilator, inhibits smooth muscle proliferation, protects against ROS, and is reduced in PH. Tetrahydrobiopterin (BH4) is a cofactor of NOS and is found to be reduced in the pulmonary arteries of patients with IPF and rescue of BH4 in the bleomycin model of rat pulmonary fibrosis and PH attenuated vascular remodeling [70]. Additionally, in vitro experiments in human pulmonary artery endothelial cells showed that increased BH4 inhibited endothelial to mesenchymal transition mirroring the role in the rat lung. Inhibition of arginase, an enzyme that produces a precursor of collagen and limits nitric oxide production, can also reduce fibrosis and PH in the neonatal rat bleomycin model [71].

HIF-1A has been identified as a transcriptional regulator of the A2B Adenosine Receptor (ADORA2B) [72]. ADORA2B is activated by adenosine, an extracellular signaling molecule that is produced by cell injury and promotes tissue repair but can promote disease progression in chronic disease states [73]. ADORA2B has been shown to play an active role in lung fibrosis [74, 75]. In the context of PH, activation of this receptor has been shown to modulate the development of PH by mediating the release of hyaluronan [76–78], from the PASMC and macrophages. Hyaluronan is a component of the lung extracellular matrix that has been implicated in the development of PH [79–81]. Activation of ADORA2B also results in increased IL-6 which is a strong mediator of Group I PH both by reducing BMPR2 and vascular endothelial growth factor receptor II (VEGFR2) [74, 82, 83]. In fact, ADORA2B receptor levels in patients with IPF directly correlated with mPAP indicating a role in the progression of PH in IPF patients [84]. In the bleomycin model of fibrosis and PH pharmacological blockage of the ADORA2B and genetic deletion of the receptor both returns pulmonary pressures to normal and reduces fibrosis. This reduction in disease state is also accompanied by reduced levels of IL-6 and ET-1 [76, 78].

An important driver of fibrosis is aberrant inflammation driven by IL-6 [85]. This role of IL-6 signaling is seen in the bleomycin model of pulmonary fibrosis. This pathway is also upregulated in human PH patients. In fact there is a direct correlation between IL-6 and increased pulmonary pressures, pulmonary artery remodeling, and RV remodeling [83, 86]. These parallel roles of IL-6 in fibrosis and PH suggest a common role of increased pulmonary inflammation in the fibrosis and PH in Group III PH.

Together these mechanisms of development of PH in IPF show that this disease progresses through complicated signaling pathways that often interact and overlap. While each mechanism offers unique potential for therapeutic intervention, this large web can complicate the study of disease progression in patients.

## 4. The Right Ventricle (RV) in IPF

An essential consequence of the development of PH is its adverse impact on the right ventricle (RV). The right ventricle (RV) is a low-pressure chamber that pumps blood to a low-pressure pulmonary circuit. It pumps the same stroke volume as its counterpart, the left ventricle (LV), with approximately 25% of the stroke work because of the low resistance and pressure of the pulmonary vasculature [94]. According to the law of Laplace, pressure and radius have a direct relationship with afterload [95]. Therefore, the initial adaptive response to pressure overload is RV hypertrophy [96–98]. Afterwards, the RV dilates in response to this pressure despite reduced fractional shortening [94]. Eventually it becomes unable to compensate and right ventricular failure occurs [99] while LV function remains preserved [100].

The term “cor pulmonale” refers to an RV alteration (structure and/or function) resulting from lung disease as long as it is not secondary to LV dysfunction [101, 102]. In chronic cor pulmonale, RVH is a direct result of chronic hypoxic pulmonary vasoconstriction and PH, leading to increased RV work and stress [103]. Additionally in IPF, fibrosis and loss of lung parenchyma also contribute to PH and RV hypertrophy by obliterating pulmonary vascular beds [104, 105] and by limiting cardiac filling secondary to relative stiffness of intrathoracic structures [106, 107]. PH is a severe complication of IPF that significantly contributes to morbidity and mortality [108] and it has been hypothesized that it is actually the RV failure component that drives the mortality in these patients [109]. This highlights the need to widen our understanding of the pathogenesis of RV failure in IPF. Little is known about the exact incidence and prevalence of cor pulmonale in IPF because right-heart catheterization (RHC), the gold standard for a definitive diagnosis [110], is an invasive procedure and these patients are considered high risk [96]. As such, most of the data regarding RHC hemodynamic information in IPF was obtained at the time of lung-transplant evaluations [111] and results may be skewed by selection bias since lung transplant is reserved for end-stage disease only.

Efforts have been made to identify RV dysfunction using noninvasive imaging methods in patients with IPF. Echocardiography on these patients is technically challenging because suboptimal images are frequently encountered [96, 112] and conventional techniques of RV longitudinal function, such as tricuspid annular plane systolic excursion (TAPSE), a parameter of global RV function and tissue Doppler systolic peak velocity, are prone to error and may not adequately reflect RV dysfunction [113]. However, RV : LV ratio [111] may be useful in predicting mortality in these patients and RV global longitudinal strain may directly correlate to FVC% and RHC-obtained mPAP as well as independently predicting functional capacity during 6-minute walking distance (6MWD) tests [114]. In the normal population, MRI is the best method for measuring RV dimensions and function; however data is still inconclusive regarding use of MRI in patients with IPF. RHC is currently the definitive diagnostic tool for RV dysfunction in IPF [110].

Clinically, cor pulmonale is a challenging diagnosis since the signs and symptoms are insensitive and nonspecific [96, 115] and distinguishing chronic lung disease from associated PH and RV failure can be difficult. Dyspnea, peripheral edema, elevated jugular venous pressure, and hepatomegaly may arise from new RV failure or progression of the underlying lung disease or a combination of both [105, 116]. In addition, physical exam may be of limited value in these patients since classical signs of RV failure (precordial heave, accentuated pulmonic component of S2, murmur of tricuspid regurgitation, and right-sided gallop) may be masked by IPF pathophysiology [105].

To complement the clinical diagnosis of cor pulmonale in IPF patients, EKG findings (rightward P-wave axis deviation, an S_1_S_2_S_3_ pattern, S_1_Q_3_ pattern, and right bundle branch block [117]) have a high specificity but very low sensitivity [96, 118]. On the other hand, 6MWD correlated better with RV failure per echochardiography [119]. Additionally, brain natriuretic peptide (BNP) seems to be a promising marker for PH and RV failure in IPF since elevated levels in serum are both a marker for PH presence in this cohort [120] and an independent predictor of prognosis [121, 122].

Mortality related to cor pulmonale is also difficult to assess; chronic lung disease is responsible for 100,000 deaths per year in the USA [123]; however the role of PH and Right-Heart Failure is not specified [96]. Cor pulmonale is the second cause of death of pulmonary hypertension worldwide [115]; however most of it is driven by COPD. Survival in PH, independent of the cause, is directly related to the capacity of the RV to adapt to the elevated pulmonary vascular load [124].

In addition to the cardiac consequences of increased pulmonary pressures, treatment for IPF may negatively impact cardiac function. Nintedanib caused cardiac events in 9.7%–10.3% of patients, 0.3–0.6% of them are fatal [125], and a three-drug combination (prednisone, azathioprine, and N-acetylcysteine) also showed cardiac adverse events in 3.9% of patients [126]; however pirfenidone demonstrated a safe cardiac profile [127–129]. Details of cardiac events were not specified. Taken together, these observations highlight our limited understating of the pathogenic RV in IPF and how current therapies for IPF may impact the heart, providing further rationale to invest in research aimed at uncovering unique mechanisms of RV failure in IPF.

## 5. Experimental Models of PH and CLD

An important difficulty in studying the development of PH in IPF is the lack of animal models that exhibit both lung fibrosis and PH. Although there are several experimental models of lung fibrosis [87, 88]m, not all of them are known to exhibit markers of PH such as increased right ventricle systolic pressure (RVSP), vascular remodeling, or elevated Fulton indices as a marker of right ventricle hypertrophy (RVH). In this review we will summarize the models that have been shown to present with features of both fibrosis and PH. The most common model of lung fibrosis uses the exposure of mice to the anticancer agent bleomycin (BLM). Typically, in this model, BLM is intratracheally instilled leading to the development of fibrosis by day 14 [88]. Alternatively, in this model, BLM is chronically administered intraperitoneally for 4 weeks resulting in the development of fibrosis [74, 89]. In both models researchers have identified hallmarks of PH including elevated RVSP, RVH, and vascular remodeling [40, 58, 76, 90]. Another model of lung fibrosis and PH is the VHL200W mice which are remarkable in that the mutation of the von Hippel-Lindau (VHL) tumor suppressor protein at codon 200 (R200W) leads to PH and lung fibrosis in mice [64]. However, these mice have been seldom used to study PH associated with lung fibrosis. Another genetic mouse model of Group III PH is the adenosine deaminase-deficient* (Ada*^−*/*−^) mice. These mice are unable to break down adenosine and as a result they develop features of chronic lung disease such as lung fibrosis and airspace enlargement [91] with hallmarks of PH [77]. Another model of fibrosis is fus-related antigen 2 (Fra-2) transgenic mice model [92]. Fra-2 is a phosphorylated protein which results in lung fibrosis and lung inflammation. The Fra-2 transgenic mice do not survive after 17 weeks of age, due to respiratory distress. These mice present with fibrotic lesions in addition to marked vascular remodeling akin to those observed in other models of lung fibrosis and PH. Unfortunately RVSP levels or RVH were not performed in these mice; thus despite marked vascular remodeling whether increased pressures are present is not known. An additional model is the transforming growth factor Beta I (TGF-*β*1) expressing a kinase-deficient human type II TGF*β* receptor (T*β*RIIdeltaK) is selectively expressed in fibroblast in a transgenic mice model used to study lung fibrosis [93]. Remarkably, exposure to these mice to chronic hypoxia results in vascular remodeling, increased RVSP, RVH, and fibrotic injury, positioning this model as a solid alternative to the bleomycin exposure models. In summary, there are limited models of PH associated with lung fibrosis which hampers our ability to identify mechanisms that are unique to the development of PH in lung fibrosis.

## 6. Present and Future Therapies for PH in IPF

Currently there is no specific therapy for PH associated with lung diseases. Treatment of the underlying disease should be optimized to reduce disease progression. Current ESC/ERS guidelines recommend that hypoxemic patients with lung disease and pulmonary hypertension should be started on long-term O_2_ therapy [130].

Several studies have analyzed the role of existing Group I PH therapies and their impact on patients with PH in the setting of IPF. In an earlier study with a small sample size and different causes of pulmonary fibrosis it was shown that inhaled prostacyclin caused a marked reduction of pulmonary pressures without affecting arterial pressure and lung diffusion [131]. Both intravenous prostaglandins and calcium channel blockers caused a significant drop of systemic pressures.

The use of sildenafil was also compared in a randomized controlled open label trial of 16 patients with PH associated with IPF that received inhaled NO (10–20 ppm) and were randomized to receive either intravenous epoprostenol or oral sildenafil. NO reduced PVR by 22%, epoprostenol by 37%, and sildenafil by 33%. However, epoprostenol increased V/Q mismatch and decreased arterial oxygenation, while NO and sildenafil maintained V/Q matching [48]. In a larger randomized controlled trial STEP-IPF trial, 180 patients received either sildenafil or placebo for 12 weeks; on a second period of 12 weeks all the placebo patients were crossed over to the sildenafil arm. There was no difference in the primary outcome (defined as an increase in 6MWD by >20%) between the two groups (10% of patients in the sildenafil group versus 7% in the placebo, *p* = 0.39); there was however improvement in the shortness of breath and quality of life questionnaires as well as small differences in arterial oxygenation and DLCO. As in the previous study there were no significant side effects [132].

Currently, there are 3 endothelin receptor antagonists (ERAs) approved for the treatment of Group I PH (bosentan, ambrisentan, and macitentan). The role of ERAs in patients with PH associated with IPF has been studied in several randomized control trials, all of which have shown no benefit from the use of these agents in patients with PH and IPF. In the BUILD-1 (Bosentan Use in Interstitial Lung Disease) trial, the safety and efficacy of the nonselective endothelin antagonist, bosentan, were studied. In this double-blind multicenter RCT of 158 patients bosentan had no superiority when compared to placebo to meet either the primary (6MWD at 1 year) or the secondary endpoint (time to death or disease progression) of the study [133]. The ARTEMIS-IPF trial, which included IPF patients with and without PH treated with the selective endothelin agonist, ambrisentan, was terminated early after an interim analysis of 492 patients demonstrated* an increase* in the disease progression in the treatment arm compared to placebo. Furthermore, patients treated with ambrisentan were more likely to develop respiratory hospitalizations, when the analysis was limited to those patients with IPF and concomitant PH results were similar [13]. Similarly the BPHIT study was a 16-week multicenter, double-blind RCT of patients diagnosed with fibrotic idiopathic pneumonia (including IPF and nonspecific interstitial pneumonia) and pulmonary hypertension treated with either bosentan or placebo. In this study the primary endpoint was a fall of 20% in the PVRi, as in previous studies there was no difference between both groups (≈28% for both arms) even when IPF-PH patients were analyzed alone [61].

Riociguat, a stimulator of the soluble guanylate cyclase, is a novel treatment for both Group I PH [134] and Group IV PH [135] (Patent-1 and CHEST-1 trials). Riociguat has also been studied in patients with fibrotic lung disease and PH. In a pilot trial by Hoeper et al. riociguat improved PVR, CO, and 6MWD [136]. However, a Phase II study was terminated by its Data Monitoring Committee due to an apparent increased in the risk for death and other serious adverse events (PH-IIP, NCT02138825).

One of the most recently approved medications for treatment of IPF in the United States is nintedanib, a nonselective inhibitor of tyrosine kinases; the INPULSIS-1 and INPULSIS-2 trials showed an improvement in FVC; however the impact on pulmonary pressures was not studied [137].

Another agent, pirfenidone an antifibrotic agent that inhibits TGF-b mediated collagen synthesis, has also been approved by the FDA for treatment of IPF after the ASCEND trial demonstrated a reduced disease progression. However as with nintedanib the impact of pulmonary pressures was not addressed [127].

Dysregulation of multiple pathways leads to the development of IPF and PH. Targeting multiple pathways with combination therapy is an attractive idea for the treatment of this condition. However, very few studies have been done to analyze the safety and efficacy of this treatment strategy in this specific patient population. The safety and pharmacokinetics of nintedanib and pirfenidone combination were studied in 50 patients; there were no serious side effects and there was a trend towards a lower exposure of nintedanib when added to pirfenidone; however if the patients had or not pulmonary hypertension was not specified in the study [138]. In a small case control study of patients with advanced IPF and PH receiving pirfenidone only or pirfenidone plus sildenafil, there was a trend towards a preserved DLCO and no difference in side effects [139]. A Phase IIb randomized controlled trial to evaluate the efficacy and safety of pirfenidone and sildenafil combination is currently recruiting patients (NCT02951429). Results from this trial could provide future directions regarding combination therapy in this very sick patient population.

Overall there are no effective PH-specific treatments in patients with IPF-PH. And as some large trials have demonstrated, pulmonary vasodilators have the potential risk of increasing the V/Q mismatch which could result in a worsening hypoxemia and disease progression. A different approach to the treatment of IPF-PH should be considered in future research studies.

The role of stem cells has not been studied in patients with PH associated with IPF. However there have been preclinical studies analyzing the role of stem/mesenchymal cell delivery in experimental models of lung fibrosis [140–143] or PH [144–146] but not both. Encouragingly, delivery of stem cells has been shown to appear to be safe in patients with either IPF [147] or PAH [148]. If regenerative therapies are able to restore endothelial function and reduce the inflammatory and profibrotic process, this modality of treatment could be a potential strategy in patients with PH associated with IPF.

In conclusion, research in PH associated with IPF can be considered an emerging field where further investigation is desperately required in order to develop novel therapies that are able to effectively target PH in IPF. Presently, therapies for PH in IPF are far from ideal and the fact that many treatments aimed at Group I PH show disappointing results in clinical trials for patients with IPF and PH highlights differential mechanisms of disease that must be uncovered. Intrinsically linked to PH, is RV failure in IPF that is also strongly linked to mortality, yet it also remains underinvestigated, with limited treatment options. PH in IPF has often been regarded as a complication of IPF and due to its lower mPAP values compared to other presentations of PH it has not received much attention and the assumption was that diminishing the extent of fibroproliferative injury would also attenuate PH hallmarks. However, our inability to effectively reverse fibrotic deposition in patients in IPF, in addition to the fact that PH and RV failure are strongly linked to mortality, should stimulate therapies that target RV failure and PH therapeutically and perhaps prophylactically too.
